# Questionnaire Survey on Enterococcus faecalis-2001 Supplementation in 137 Patients With Oral Diseases at Two Facilities

**DOI:** 10.7759/cureus.91382

**Published:** 2025-09-01

**Authors:** Masaki Minabe, Sari Ueno, Kasumi Hata, Yuria Akira, Nana Morita, Homare Kawachi, Katsuhiko Sakai, Kohei Takahashi, Michiyoshi Kouno, Takeshi Nomura

**Affiliations:** 1 Department of Oral Oncology, Oral and Maxillofacial Surgery, Tokyo Dental College, Ichikawa, JPN; 2 Department of Oral Medicine and Hospital Dentistry, Tokyo Dental College, Ichikawa, JPN; 3 Department of Dentistry and Oral Surgery, Ushiku Aiwa General Hospital, Ushiku, JPN; 4 Department of Oral Medicine and Hospital Dentistry, Tokyo Dental College, Chiba, JPN; 5 Department of Pharmacology, School of Pharmacy, International University of Health and Welfare, Ohtawara, JPN; 6 Department of Dermatology, Tokyo Dental College Ichikawa General Hospital, Ichikawa, JPN

**Keywords:** enterococcus faecalis 2001, gut–oral axis, lactic acid bacteria, oral diseases, questionnaire survey

## Abstract

Objective: Lactic acid bacteria are gaining attention not only for their role in regulating the intestinal flora but also for their immunostimulatory effects. Recent clinical trials and in vitro studies have demonstrated the efficacy of heat-killed *Enterococcus faecalis* 2001 (EF-2001) in treating oral candidiasis, suggesting its potential to improve oral microbial flora. However, its effectiveness in other oral conditions remains unclear. This study aimed to evaluate the clinical utility of EF-2001 in a broader range of oral diseases.

Methods: A prospective observational questionnaire-based study was conducted on 137 patients who had used granules or paste containing 7.5×10¹¹ EF-2001. Data were collected from November 2020 to March 2021 and from July 2021 to August 2024 at two different facilities.

Results: At facility one (n=38), oral diseases included xerostomia (n=10, 26%), glossitis (n=8, 21%), stomatitis (n=7, 18%), oral candidiasis (n=5, 13%), and oral lichen planus (n=5, 13%). Ten patients were receiving cancer treatment or undergoing perioperative oral care. Intestinal symptoms were reported in 34 patients, mainly constipation (n=27, 71%) and diarrhea (n=7, 18%). Improvement in oral symptoms and intestinal symptoms was observed in 25 of 35 respondents (71%) and 18 of 31 (58%), respectively. The most common time-to-symptom improvement was two to four weeks. A significant association was found between improvements in oral and intestinal symptoms.

At facility two (n=99), oral diseases included oral lichen planus (OLP) (n=49, 49%), glossodynia (n=12, 12%), oral candidiasis (n=10, 10%), pemphigus vulgaris (n=6, 6%), and Sjögren’s syndrome (n=6, 6%). Intestinal symptoms were reported in 67 patients, mainly constipation (n=51, 52%) and diarrhea (n=16, 16%). Improvement in oral symptoms was reported in 64 of 98 respondents (65%), and in intestinal symptoms in 42 of 65 (65%). The most frequent time-to-improvement was two to four weeks for oral symptoms and four to eight weeks for intestinal symptoms. A significant association was again observed between improvements in oral and intestinal symptoms.

Conclusion: Supplementation with EF-2001 resulted in subjective improvements in both oral and intestinal symptoms across various oral diseases, with a notable association between the two types of symptom improvement.

## Introduction

Lactic acid bacteria have gained increasing attention for their immunostimulatory properties and their ability to modulate intestinal function by regulating the gut microbiota [1,2]. These bacteria are generally classified into three categories: probiotics, prebiotics, and biogenics. While probiotics, such as bifidobacteria, are widely recognized, they are mostly degraded by gastric acid after ingestion. Even if some survive transit to the intestine, they rarely colonize and are typically excreted as transient flora. Prebiotics, including oligosaccharides and dietary fibers, act as nutrients for beneficial bacteria, thereby increasing their populations.

Biogenics, on the other hand, include heat-killed strains such as *Enterococcus faecalis *2001 (EF-2001), which is thought to possess potent immunostimulatory activity [3]. The EF-2001 is prepared by heat-treating the bacterium to extract bioactive cell wall components rich in β-glucans, which further enhances its immunostimulatory capacity. It functions as a biological response modifier (BRM) [4], and upon reaching Peyer’s patches in the small intestine, it stimulates the production of immunoglobulin A (IgA) [3,5]. It promotes immune function and enhances the population of beneficial gut microbes. The key immunoactive elements are polysaccharides and peptide glucans found in the bacterial cell wall, which serve as immune cell activators. Indeed, leukocyte activity assays have shown the highest activity in the cell wall fraction, while the cytoplasm and nucleus showed minimal activity [5,6].

A substantial body of research supports the therapeutic potential of EF-2001, including its anti-allergic, anti-inflammatory, anti-tumor, antioxidant, and anti-sarcopenic effects [7-9]. More recently, the EF-2001 has been shown to suppress inflammatory cytokine production in animal models of inflammatory bowel disease (IBD), thereby alleviating symptoms of IBD [10]. These findings have also drawn attention to the gut-brain axis as a potential mechanism of action.

In the oral field, both in vitro and clinical studies have explored the use of EF-2001. Laboratory studies have demonstrated that EF-2001 adheres to *Candida albicans* and *Candida glabrata*, inhibiting their proliferation. Clinical studies have reported that 92% of participants experienced a reduction in oral Candida counts and symptomatic relief. Additional studies have noted improvements in subjective symptoms such as cooling sensations and mental health, suggesting possible systemic effects [11]. Given this background, the EF-2001 has been used in patients with oral candidiasis, xerostomia, oral diseases accompanied by intestinal symptoms, and allergic oral mucosal disorders.

In this study, we conducted a prospective observational study focusing on patient-reported outcomes after EF-2001 supplementation. The EF-2001 is a commercially available food supplement that was recommended during routine oral medicine practice, and patients who wished to take it provided written informed consent to participate in the questionnaire survey. The structured questionnaire was administered approximately three months after initiation, and both oral and intestinal symptoms were evaluated.

## Materials and methods

Subjects

At facility one (Department of Dentistry and Oral Surgery, Ushiku Aiwa General Hospital, Ushiku, JPN), 38 patients who received EF-2001 supplementation between November 2020 and March 2021 were enrolled. At facility two (Department of Oral and Maxillofacial Surgery, Tokyo Dental College Ichikawa General Hospital, Ichikawa, JPN), 99 patients who received EF-2001 supplementation between July 2021 and August 2024 were enrolled. All patients who met the eligibility criteria during the study period and provided consent were included; no a priori sample size calculation was performed, as this was an exploratory study. Outpatients with oral diseases who were recommended EF-2001 as part of routine clinical care and who agreed to participate in the survey were included in the study. Those with the inability to complete the questionnaire due to cognitive decline, refusal to participate, or incomplete consent documentation were excluded from the study.

EF-2001 supplementation

The EF-2001 supplement used in this study was either a granule or paste formulation, each containing 7.5 × 10¹¹ cells of heat-killed EF-2001 (patent no. 3151442), manufactured by Nihon Belm Co. Ltd. (Suita City, Osaka, JPN). The paste formulation additionally contained starch hydrolysate (1,5-anhydrofructose). Other excipients were not considered relevant to this study and are therefore not listed in detail. Patients were instructed to take one packet before bedtime, dissolving the granules in a small amount of water or placing the paste directly in the mouth, holding the preparation briefly in the oral cavity before swallowing. This administration method was chosen because EF-2001 is thought to be better absorbed at night, when oral dryness is more pronounced, and may exert a direct effect on the oral cavity. The choice of dosage form (granule or paste) was based on patient preference.

Data collection and statistical analysis

A structured questionnaire, independently developed for this study, was administered approximately three months after the initiation of EF-2001 supplementation. The questionnaire assessed oral symptoms (xerostomia, stomatitis, glossodynia, dysgeusia, tongue coating, halitosis, and other comments) and intestinal symptoms (constipation, diarrhea) prior to EF-2001 supplementation, with multiple responses allowed. Patients were then asked to evaluate perceived improvements in oral symptoms, intestinal symptoms, and overall condition (the latter in facility one only). Responses regarding improvement of intestinal symptoms were collected only from patients who reported such symptoms. Response options were “improved,” “slightly improved,” “no change,” “slightly worse,” and “worse.” The duration until improvement was categorized as one to two weeks, two to four weeks, four to eight weeks, or eight to 12 weeks. Patients were also asked about their willingness to continue EF-2001 (yes, somewhat, neutral, not really, no). Additional data, including sex, age, oral disease diagnosis, systemic diseases, and intestinal symptoms, were extracted from medical records. Associations between oral symptoms, intestinal symptoms, and general condition were analyzed using Fisher’s exact test. A p-value < 0.05 was considered statistically significant.

Ethical considerations

This study was approved by the Institutional Review Boards of Ushiku Aiwa General Hospital (approval no. UA20210326) and Tokyo Dental College Ichikawa General Hospital (approval no. I 21-33). All patients received both oral and written explanations and provided written informed consent prior to participation.

## Results

Subject characteristics

Facility One

Among the 38 participants, 10 were male and 28 were female, with a mean age of 74 years. The EF-2001 granules and paste were used in 16 and 23 cases, respectively (one patient used both dosage forms). The oral diseases included xerostomia (n = 10, 26%), glossitis (n = 8, 21%), stomatitis (n = 7, 18%), oral candidiasis (n = 5, 13%), oral lichen planus (OLP) (n = 5, 13%), neuropathic pain (n = 2, 5%), and jaw osteonecrosis (n = 1, 3%). Ten patients received perioperative oral management. Systemic conditions included malignant tumors (n = 11, 29%), hypertension (n = 11, 29%), gastrointestinal disorders (n = 5, 13%), rheumatoid arthritis (n = 3, 8%), and diabetes mellitus (n = 3, 8%). Intestinal symptoms were also reported: diarrhea (n = 7, 20%) and constipation (n = 28, 80%) (Table 1).

**Table 1:** Demographic and clinical characteristics of patients at facility one The data are presented as n and %. *EF-2001 granules and paste were used; one case involved the use of both forms. **Others include oral diseases observed in 1 to 3 cases (e.g., Sjogren syndrome, glossodynia, dysgeusia; n=18). *** Others include systemic diseases observed in 1 to 3 cases (e.g., dyslipidemia, osteoporosis, asthma; n=35). Mean age = 74 years. OLP: Oral lichen planus, EF-2001: *Enterococcus faecalis* 2001

Variable		N (cases)	Percentage
Gender	Male	10	26
	Female	28	74
Dosage form	Granule*	16	42
	Paste*	23	61
Oral diseases (with duplication)	Xerostomia	10	26
	Glossitis	8	21
	Stomatitis	7	18
	Oral candidiasis	5	13
	OLP	5	13
	Neuropathic pain	2	5
	Osteonecrosis of the jaw	1	3
	Others**	30	79
Systemic diseases (with duplication)	Malignant tumor	11	29
	Hypertension	11	29
	Gastrointestinal disease	5	13
	Cardiovascular disease	4	11
	Rheumatoid arthritis	3	8
	Diabetes mellitus	3	8
	Others***	50	132

Facility Two

Of the 99 participants, 19 were male and 80 were female, with a mean age of 67 years. The EF-2001 granules and paste were used in 24 and 75 cases, respectively. Diagnoses included OLP (n = 49, 49%), glossitis (n = 12, 12%), oral candidiasis (n = 10, 10%), pemphigus vulgaris (n = 6, 6%), Sjögren’s syndrome (n = 6, 6%), xerostomia (n = 5, 5%), pemphigoid (n = 5, 5%), leukoplakia (n = 4, 4%), taste disorders (n = 4, 4%), granulomatous cheilitis (n = 3, 3%), stomatitis (n = 3, 3%), and zinc deficiency (n = 2, 2%). Systemic diseases included hypertension (n = 26, 26%), dyslipidemia (n = 12, 12%), osteoporosis (n = 11, 11%), diabetes mellitus (n = 8, 8%), hay fever (n = 8, 8%), metal allergy (n = 5, 5%), cataracts (n = 4, 4%), and gastroesophageal reflux disease (n = 4, 4%). Intestinal symptoms included diarrhea (n = 16, 16%) and constipation (n = 51, 52%) (Table 2).

**Table 2:** Demographic and clinical characteristics of patients at facility two The data are presented as n and %. *Others include oral diseases observed in one to three cases (e.g., bisphosphonate-related osteonecrosis of the jaw (BRONJ), osteonecrosis of the jaw, neuropathic pain; n=10). **Others include systemic diseases observed in one to three cases (e.g., angina pectoris, hyperuricemia, rheumatoid arthritis; n=70). Mean age: = 67 years. OLP: Oral lichen planus, EF-2001: *Enterococcus faecalis* 2001

Variable		N (cases)	Percentage
Gender	Male	19	19
	Female	80	81
Dosage form	Granule	24	24
	Paste	75	76
Oral diseases (with duplication)	OLP	49	49
	Glossitis	12	12
	Oral candidiasis	10	10
	Pemphigus vulgaris	6	6
	Sjögren's syndrome	6	6
	Xerostomia	5	5
	Pemphigoid	5	5
	Leukoplakia	4	4
	Taste disorders	4	4
	Granulomatous cheilitis	3	3
	Stomatitis	3	3
	Zinc deficiency	2	2
	Others*	10	10
Systemic diseases (with duplication)	Hypertension	26	26
	Dyslipidemia	12	12
	Osteoporosis	11	11
	Diabetes mellitus	8	8
	Hay fever	8	8
	Metal allergy	5	5
	Cataracts	4	4
	Gastroesophageal reflux disease	4	4
	Others**	89	90

Questionnaire results (dosage form not distinguished)

Facility One

Oral symptoms reported included xerostomia (n = 15, 39%), stomatitis (n = 11, 29%), glossodynia (n = 8, 21%), dysgeusia (n = 6, 16%), tongue coating (n = 5, 13%), halitosis (n = 4, 11%), gingival pain (n = 2, 5%), and sticky saliva (n = 1, 3%). Intestinal symptoms included constipation (n = 27, 71%), diarrhea (n = 7, 18%), and normal (n = 7, 18%). Improvement in oral symptoms was reported in 25 cases: eight (21%) “improved” and 17 (45%) “slightly improved,” while 10 (26%) reported “no change.” The most frequent time to improvement was two to four weeks (n = 9, 38%), followed by one to two weeks (n = 7, 29%) and four to eight weeks (n = 6, 25%). Improvement in intestinal symptoms was reported in 18 cases: nine (29%) “improved,” nine (29%) “slightly improved,” 12 (39%) “no change,” one (3%) “slightly worse,” and zero (0%) “worse.” For intestinal symptoms, time to improvement was two to four weeks (n = 8, 44%), one to two weeks (n = 6, 33%), and 4-8 weeks (n = 4, 22%).

Regarding general condition, improvement was reported in 19 out of 38 cases (50%) (n = 9, 47% “improved”; n = 10, 53% “slightly improved”), while 19 (50%) reported “no change” and one (3%) “slightly worse.” Future willingness to continue EF-2001 was expressed by 28 patients. Of whom, 12 (33%) responded “yes,” 16 (44%) said “a little,” four (11%) stated “neither,” three (8%) responded “not much,” and one (3%) said “no.” Reasons included feeling better overall (n = 4, 14%), oral freshness (n = 3, 11%), relief of thirst (n = 3, 11%), good taste (n = 2, 7%), and improved bowel movements (n = 2, 7%) (Table 3).

**Table 3:** Self-reported changes in oral and intestinal symptoms after EF-2001 supplementation in facility one The data are presented as n and %. *Intestinal symptoms were observed in 31 patients; three patients reported both constipation and diarrhea. **Non-responders: n = 3 for improvement in oral symptoms; n = 1 for duration until improvement of oral symptoms; n = 2 for willingness to continue use. The percentages were calculated based on the number of respondents. EF-2001: *Enterococcus faecalis* 2001

Parameters	Results	N (cases)	Percentage
Oral symptoms	Xerostomia	15	39
	Stomatitis	11	29
	Glossodynia	8	21
	Dysgeusia	6	16
	Tongue coating	5	13
	Halitosis	4	11
	Gingival pain	2	5
	Sticky saliva	1	3
Intestinal symptoms	Constipation	27*	71
	Diarrhea	7*	18
	Normal	7	18
Improvement in oral symptoms**	Improved	8	23
	Slightly improved	17	49
	No change	10	29
	Slightly worse	0	0
	Worse	0	0
Improvement in intestinal symptoms	Improved	9	29
	Slightly improved	9	29
	No change	12	39
	Slightly worse	1	3
	Worse	0	0
Duration until improvement of oral symptoms**	1–2 weeks	7	29
	2–4 weeks	9	38
	4–8 weeks	6	25
	8-12 weeks	2	8
Duration until improvement of intestinal symptoms	1–2 weeks	6	33
	2–4 weeks	8	44
	4–8 weeks	4	22
	8-12 weeks	0	0
Willingness to continue use**	Yes	12	33
	A little	16	44
	Neither	4	11
	Not much	3	8
	No	1	3

Improvements were seen in over 70% of oral symptoms and approximately 50% of intestinal and general symptoms. Fisher’s exact test showed statistically significant associations between improvement in oral and intestinal symptoms (p = 0.001) (Table 4), oral symptoms and general condition (p = 0.001) (Table 5), and intestinal symptoms and general condition (p < 0.001) (Table 6).

**Table 4:** Association between improvement in oral and intestinal symptoms at facility one Fisher’s exact test; p = 0.001

Parameters	No improvement of intestinal symptoms	Improvement of intestinal symptoms	Total
No improvement of oral symptoms	9	0	9
Improvement of oral symptoms	8	16	24
Total	17	16	33

**Table 5:** Association between improvement in oral symptoms and general condition at facility one Fisher’s exact test; p = 0.001

Parameters	No improvement in general condition	Improvement of general condition	Total
No improvement of intestinal symptoms	10	0	10
Improvement of intestinal symptoms	9	16	25
Total	19	16	35

**Table 6:** Association between improvement in intestinal symptoms and general condition at facility one Fisher’s exact test; p < 0.001

Parameter	No improvement of intestinal symptoms	Improvement of intestinal symptoms	Total
No improvement of oral symptoms	16	2	18
Improvement of oral symptoms	4	15	19
Tota	20	17	37

Facility Two

Reported oral symptoms included xerostomia (n = 44, 45%), glossodynia (n = 36, 37%), stomatitis (n = 34, 35%), tongue coating (n = 28, 29%), halitosis (n = 19, 19%), gingival pain (n = 18, 18%), sticky saliva (n = 14, 14%), and dysgeusia (n = 11, 11%). Intestinal symptoms included constipation (n = 51, 52%), diarrhea (n = 16, 16%), and normal (n = 35, 35%). Improvement in oral symptoms was reported in 64 cases: 27 (27%) “improved” and 37 (37%) “slightly improved,” while 35 (35%) reported “no change.” Time to improvement was most often two to four weeks (n = 19, 30%), followed by four to eight weeks (n = 15, 23%), eight to 12 weeks (n = 14, 22%), one to two weeks (n = 11, 17%), and >12 weeks (n = 5, 8%). Improvement in intestinal symptoms was seen in 42 cases: 29 (45%) “improved,” 13 (20%) “slightly improved,” 22 (34%) “no change,” 0 (0%) “slightly worse,” and 0 (0%) “worse.” Time to improvement was four to eight weeks (n = 15, 36%), two to four weeks (n = 13, 31%), eight to 12 weeks (n = 7, 17%), one to two weeks (n = 6, 14%), and >12 weeks (n = 1, 2%).

Among 98 respondents, willingness to continue EF-2001 was high: 39 (40%) responded “yes,” 40 (41%) said “a little,” nine (9%) stated “neither,” nine (9%) expressed “not much,” and one (1%) responded “no.” Reasons included improvement in oral symptoms (n = 38, 48%), intestinal symptoms (n = 26, 33%), and general condition (n = 7, 9%). Specific oral improvements included dry mouth (n = 4, 5%), halitosis and OLP (n = 3, 4% each), gingival condition and saliva stickiness (n = 2, 3% each), and tongue pain, tongue coating, and oral candidiasis (n = 1, 1% each) (Table 7). Overall, 66% experienced oral symptom improvement, and approximately 70% experienced improvement in intestinal symptoms. Fisher’s exact test showed a statistically significant association between oral and intestinal symptom improvement (p = 0.022) (Table 8).

**Table 7:** Self-reported changes in oral and intestinal symptoms after EF-2001 supplementation in facility two The data are presented as n and %. *Intestinal symptoms were observed in 67 patients; three patients reported both constipation and diarrhea. **Non-responders: n = 1 for willingness to continue use. The percentages were calculated based on the number of respondents. EF-2001: *Enterococcus faecalis *2001

Parameters	Results	N (cases)	Percentage
Oral symptoms	Xerostomia	44	44
	Glossodynia	36	36
	Stomatitis	34	34
	Tongue coating	28	28
	Halitosis	19	19
	Gingival pain	18	18
	Dysgeusia	11	11
	Sticky saliva	14	14
Intestinal symptoms	Constipation	51*	52
	Diarrhea	16*	16
	Normal	35	35
Improvement in oral symptoms	Improved	27	27
	Slightly improved	37	37
	No change	35	35
	Slightly worse	0	0
	Worse	0	0
Improvement in intestinal symptoms	Improved	13	20
	Slightly improved	29	45
	No change	22	34
	Slightly worse	0	0
	Worse	0	0
Duration until improvement of oral symptoms	1–2 weeks	11	17
	2–4 weeks	19	30
	4–8 weeks	15	23
	8-12 weeks	14	22
	12- weeks	5	8
Duration until improvement of intestinal symptoms	1–2 weeks	6	14
	2–4 weeks	13	31
	4–8 weeks	15	36
	8-12 weeks	7	17
	12- weeks	1	2
Willingness to continue use**	Yes	39	40
	A little	40	41
	Neither	9	9
	Not much	9	9
	No	1	1

**Table 8:** Association between improvement in oral and intestinal symptoms at facility two Fisher’s exact test; p = 0.022

Parameters	No improvement of intestinal symptoms	Improvement of intestinal symptoms	Total
No improvement of oral symptoms	16	6	22
Improvement of oral symptoms	19	26	45
Total	35	32	67

Focus on OLP cases

Among participants with OLP (n = 49), reported oral symptoms included stomatitis (n = 23, 47%), xerostomia (n = 17, 35%), gingival pain (n = 11, 22%), tongue coating (n = 10, 20%), halitosis (n = 7, 14%), glossodynia (n = 6, 12%), sticky saliva (n = 3, 6%), and dysgeusia (n = 2, 4%). Intestinal symptoms included constipation (n = 25, 51%), diarrhea (n = 8, 16%), and normal (n = 16, 33%). Oral symptoms improved in 28 cases: 12 (25%) “improved” and 16 (33%) “slightly improved.” Intestinal symptoms improved in 25 cases: 5 (15%) “improved” and 20 (61%) “slightly improved.” Time to improvement in oral symptoms was most often two to four weeks, while that of intestinal symptoms was also most often two to four weeks (Table 9).

**Table 9:** Self-reported changes in oral and intestinal symptoms after EF-2001 supplementation in OLP patients The data are presented as n and %. *Non-responders: n=1 for improvement in oral symptoms; n=1 for duration until improvement of oral symptoms. The percentages were calculated based on the number of respondents. EF-2001: Enterococcus faecalis 2001, OLP: Oral lichen planus

Parameters	Results	N (cases)	Percentage
Oral symptoms	Stomatitis	23	47
	Xerostomia	17	35
	Gingival pain	11	22
	Tongue coating	10	20
	Halitosis	7	14
	Glossodynia	6	12
	Sticky saliva	3	6
	Dysgeusia	2	4
Intestinal symptoms	Constipation	25	51
	Diarrhea	8	16
	Normal	16	33
Improvement in oral symptoms	Improved	12	25
	Slightly improved	16	33
	No change	19	40
	Slightly worse	1	2
	Worse	0	0
Improvement in intestinal symptoms	Improved	5	15
	Slightly improved	20	61
	No change	8	24
	Slightly worse	0	0
	Worse	0	0
Duration until improvement of oral symptoms	1–2 weeks	5	19
	2–4 weeks	8	30
	4–8 weeks	5	19
	8-12 weeks	6	22
	12- weeks	3	11
Duration until improvement of intestinal symptoms	1–2 weeks	1	4
	2–4 weeks	17	68
	4–8 weeks	3	12
	8-12 weeks	2	8
	12- weeks	2	8
Willingness to continue use	Yes	20	41
	A little	15	31
	Neither	3	6
	Not much	4	8
	No	1	2

Fisher’s exact test showed a significant association between oral and intestinal symptom improvement in OLP cases (p = 0.011) (Table 10). Among patients with improved intestinal symptoms, 75% (21/28) also reported improved oral symptoms, compared to only 33% (7/21) among those with no improvement in intestinal symptoms (Table 11).

**Table 10:** Association between improvement in oral and intestinal symptoms among OLP patients Fisher’s exact test; p=0.0078; OLP: Oral lichen planus

Parameters	No improvement of intestinal symptoms	Improvement of intestinal symptoms	Total
No improvement of oral symptoms	14	7	21
Improvement of oral symptoms	7	21	28
Total	21	28	49

**Table 11:** Association between improvement in intestinal symptoms and concurrent improvement in oral symptoms among OLP patients OLP: Oral lichen planus

Improvement in intestinal symptoms	Cases with improved oral symptoms	Total cases	Percentage
Yes	21	28	75
No	7	21	33

Questionnaire results by dosage form

The improvement rates differed slightly between dosage forms. In facility one, the paste group showed higher improvement rates than the granule group for both oral (75% vs. 66%) and intestinal symptoms (60% vs. 35%), as well as a higher willingness to continue treatment (82% vs. 69%). In facility two, the oral and intestinal symptom improvement rates were similar between the two dosage forms (oral: 68% paste vs. 54% granule; intestinal: 69% vs. 73%), though willingness to continue was slightly higher in the granule group (96% vs. 75%). These findings suggest that paste may be more effective in older patients with perioperative management, while both forms may be beneficial depending on the patient’s condition. A detailed breakdown by dosage form is provided in Appendix A.

Case report on concurrent improvement of ulcerative colitis and OLP with EF-2001

**Figure 1:**
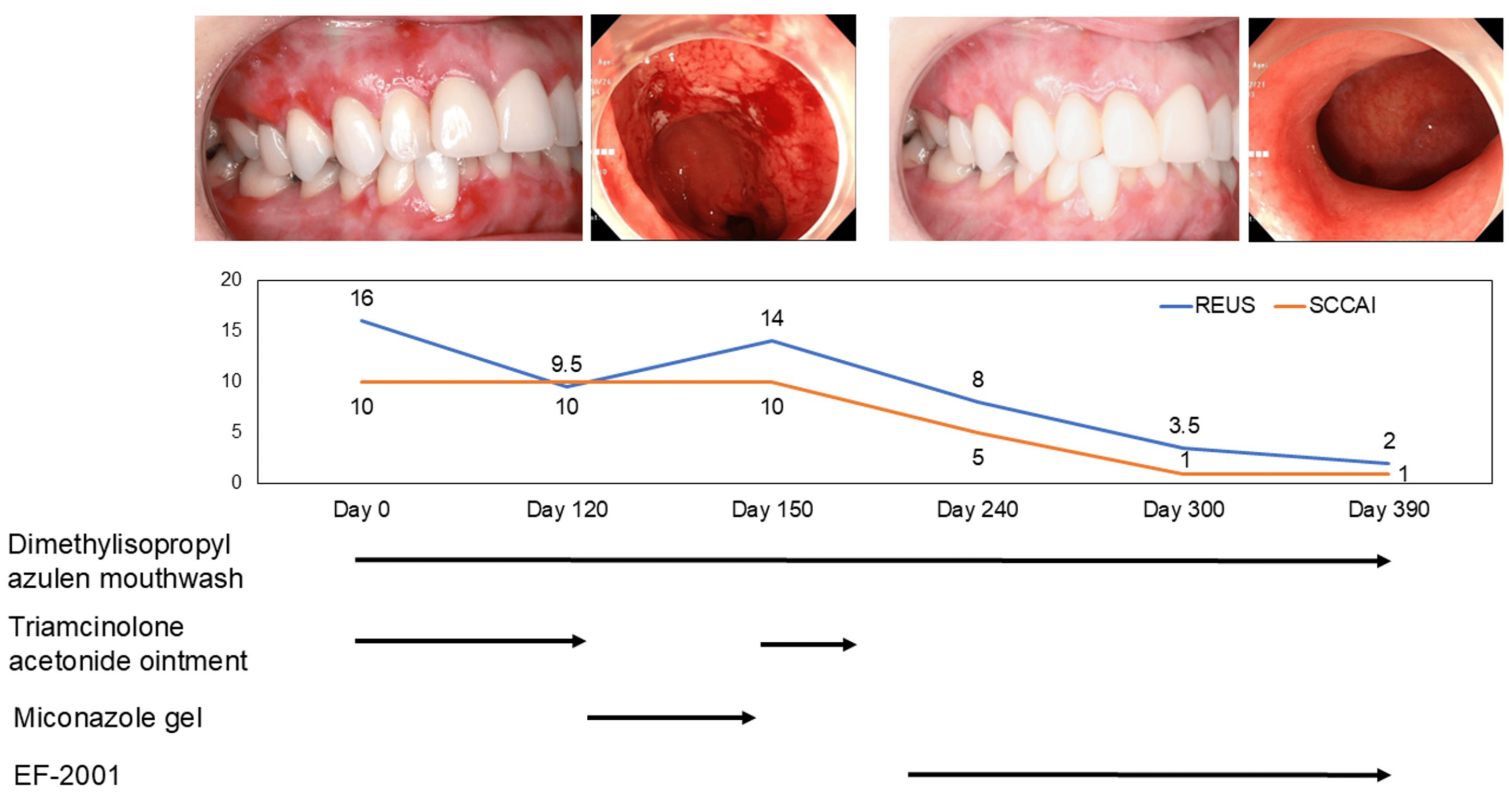
Clinical course and treatment timeline of a patient with OLP and UC This figure illustrates the clinical progression, treatment interventions, and endoscopic findings over time. The EF-2001 administration was initiated on day 210 of illness. Hematochezia persisted during the first month but resolved by the second month. Follow-up endoscopy confirmed healing of colonic ulcers, despite no changes to the patient’s UC medication regimen. Concurrently, OLP symptoms and halitosis improved markedly. Objective scoring showed a strong positive correlation between OLP severity (reticulation/keratosis, erythema, and ulceration (REU) score) and UC activity (simple clinical colitis activity index (SCCAI)). Spearman’s rank correlation coefficient = 0.926, p = 0.008 OLP: Oral lichen planus, UC: Ulcerative colitis

The relationship between OLP and intestinal symptoms remains unclear, although OLP is recognized as an extraintestinal manifestation of ulcerative colitis (UC). We report a case in which both conditions improved following EF-2001 paste supplementation. A 40-year-old woman presented with generalized gingival erythema and was diagnosed with OLP via biopsy. She had a history of UC, for which she was taking mesalazine and budesonide, but continued to experience frequent bowel movements, urgency, and hematochezia. For OLP, she used triamcinolone acetonide ointment, which led to secondary oral candidiasis. After antifungal treatment with miconazole, steroid therapy was resumed but remained ineffective. The EF-2001 in paste form was initiated to prevent candidiasis and manage OLP. One month later, gingival erythema was reduced, and by three months, mucosal healing was nearly complete. Notably, her intestinal symptoms also improved, despite no changes in UC medications. A strong association was found between OLP and UC symptom scores (Spearman’s ρ = 0.926, p = 0.008) (Figure 1). Halitosis, previously attributed to UC, also resolved.

## Discussion

At facility one, the majority of participants were elderly patients undergoing perioperative oral management, many of whom had malignant tumors. The most frequently observed oral diseases were xerostomia, glossitis, stomatitis, oral candidiasis, and OLP. In the questionnaire, the most commonly reported oral symptom was dry mouth, followed by stomatitis, glossodynia, dysgeusia, tongue coating, and halitosis. Additionally, over 80% of participants reported intestinal symptoms such as constipation or diarrhea. These trends may reflect the advanced age and clinical conditions of the participants.

Facility two did not include perioperative cases and encompassed a broader range of oral medicine disorders, with OLP being the most common diagnosis. As in facility one, dry mouth was the most frequent symptom, followed by glossodynia and stomatitis. Intestinal symptoms were present in 68% of patients, though slightly less frequent than in facility one, possibly due to differences in age, comorbidities, or treatment backgrounds.

The EF-2001 supplementation was associated with high rates of symptom improvement in both oral and intestinal conditions (oral: 71% at facility one, 65% at facility two; intestinal: 58% at facility one, 65% at facility two; general condition: 50% at facility one). These findings suggest that EF-2001 may support the improvement of oral and intestinal symptoms, particularly in patients with underlying dysbiosis. The high rate of participants expressing a desire to continue EF-2001 likely reflects these perceived benefits. While improvement rates for intestinal symptoms were lower at facility one, this may be due to the high proportion of cancer patients with treatment-induced gastrointestinal complications. Conversely, the slightly lower improvement in oral symptoms at facility two may relate to the multifactorial nature of oral medicine disorders. Aging is also associated with a decline in beneficial gut flora, increased pathogenic bacteria, and immune dysfunction, which may contribute to malignancy. Lactic acid bacteria, including EF-2001, may help counter these changes by stimulating intestinal immunity via Peyer’s patches and improving microbial balance [3].

In facility one, significant associations were found among improvements in oral, intestinal, and general symptoms. This suggests that EF-2001’s effects on oral and intestinal symptoms may be interconnected and related to systemic health. Many patients in facility one had concurrent xerostomia and constipation, likely due to cancer treatment-related dysbiosis and immune modulation, which may have increased their responsiveness to EF-2001. Facility two also demonstrated a significant association between oral and intestinal improvements. These findings support the concept that immune-mediated dysbiosis in the oral and gut microbiota may be mutually influential in oral mucosal diseases. Oral lichen planus is known to be associated with UC [12], and oral and intestinal dysbiosis has also been reported in autoimmune blistering disorders, suggesting microbiome involvement in their pathogenesis [13,14].

Regarding the different dosage forms, granules were more commonly used by patients with stomatitis, whereas paste was more frequently chosen by those with xerostomia. The paste formulation contains 1,5-anhydrofructose (1,5-AF), which has been reported to suppress plaque formation and inhibit the growth of *Streptococcus mutans*, a major causative agent of dental caries [15]. It also exhibits anti-inflammatory and antioxidant properties. In facility one, patients using the paste formulation showed higher rates of improvement in both oral (75% vs. 66%) and intestinal symptoms (60% vs. 35%) compared to those using granules (see Appendix A). In contrast, in facility two, the improvement rates for oral symptoms were 68% with paste and 54% with granules, while the rates for intestinal symptoms were 67% with paste and 60% with granules. These findings suggest that paste may be more effective in older patients or those undergoing chemotherapy, such as those at facility one, who commonly experience both xerostomia and constipation. The observed difference was especially notable for intestinal symptoms in facility one. Additionally, a higher proportion of participants in facility one expressed a desire to continue using EF-2001 paste (82%) compared to granules (69%), which may reflect greater subjective improvement. Although improvement rates were broadly similar between formulations in facility two, willingness to continue was still more frequent with paste (75%) than with granules (96%). The greater efficacy of paste may be partially explained by its retention time in the oral cavity, allowing prolonged contact with the oral microbiota. Furthermore, the active ingredient 1,5-AF may contribute to improvements in both oral and intestinal environments.

Regarding the relationship between UC and OLP, UC is a chronic inflammatory bowel disease that often involves extraintestinal manifestations in 16.7% to 40% of patients [16]. Oral symptoms of UC include both disease-specific (e.g., pyostomatitis vegetans) and nonspecific features such as OLP, halitosis, periodontal disease, taste disturbances, xerostomia, and aphthous ulcers [13]. Although the etiology of UC is not fully understood, dysregulated immune responses to the intestinal microbiota are thought to play a key role [17]. Fecal microbiome studies have shown a decrease in Lachnospiraceae and reduced butyrate production in UC patients [18]. In a UC mouse model, EF-2001 administration alleviated diarrhea and mucosal bleeding by suppressing proinflammatory cytokine overproduction [19]. In the present case, a patient with both UC and OLP showed simultaneous improvement in gastrointestinal and oral symptoms, including halitosis, after EF-2001 administration. This suggests a potential link between UC and OLP and supports the hypothesis that improving intestinal symptoms may positively influence oral disease activity.

This study has several limitations. First, it relied on a questionnaire-based design, depending on subjective patient-reported outcomes, which may introduce recall bias and variability in symptom interpretation. Second, the questionnaire was not validated, limiting the reliability of the findings. Third, the study lacked a placebo control group, preventing causal inference. Fourth, the study population was limited to patients who agreed to use EF-2001, raising the possibility of selection bias. Fifth, the sample size for some oral conditions was small, making subgroup analysis difficult. Sixth, the follow-up period was relatively short, and the long-term effects remain unclear. Finally, objective biological assessments (e.g., salivary flow rate, photographic records, or oral and gut microbiome analyses) were not included. Incorporating such measures into future prospective studies will be essential to further elucidate the mechanisms underlying the observed improvements.

## Conclusions

The EF-2001 supplementation led to symptom improvement in various oral medicine conditions, often in conjunction with improvement in intestinal symptoms. These findings suggest a potential therapeutic role for EF-2001 in modulating both oral and gut microbiota, particularly in patients with dysbiosis-related oral diseases. Future studies with larger cohorts and objective outcome measures are warranted to confirm these benefits and clarify the underlying mechanisms.

## Appendices

Questionnaire results (by dosage form)

Facility One

Granule type: Oral symptoms reported by patients using the granule type (multiple responses allowed) included stomatitis (n=6, 38%), xerostomia (n=4, 25%), dysgeusia (n=4, 25%), glossodynia (n=3, 19%), tongue coating (n=1, 6%), halitosis (n=1, 6%), gingival pain (n=1, 6%), and sticky saliva (n=1, 6%). Stomatitis was the most frequently reported symptom. Intestinal symptoms included constipation (n=9, 56%) and diarrhea (n=3, 19%); five (31%) were normal. The overall improvement rate in oral symptoms was 73% (three (20%) “improved,” eight (53%) “slightly improved,” five (33%) “no change,” 0 (0%) “slightly worse,” and zero (0%) “worse”). Time to improvement was reported as one to two weeks (n = 3, 21%), two to four weeks (n = 4, 29%), four to eight weeks (n = 2, 14%), and eight to 12 weeks (n = 1, 7%).

The improvement rate in intestinal symptoms was 45% (three (27%) “improved,” two (18%) “slightly improved,” six (55%) “no change,” zero (0%) “slightly worse,” and zero (0%) “worse”) with most improvements occurring within two to four weeks. Reported time to improvement was one to two weeks (n = 3, 60%), two to four weeks (n = 1, 20%), four to eight weeks (n = 1, 20%), and two to four months (n = 0, 0%). Improvement in general condition was reported in 47% of participants (20% “improved,” 27% “slightly improved,” 47% “no change,” 7% “slightly worse,” and 0% “worse”). Regarding willingness to continue treatment, 69% expressed interest (five (36%) said “yes,” five (36%) said “a little,” two (14%) stated “neither,” two (14%) said “not much,” and none (0%) said “no”) (Table 12).

**Table 12:** Self-reported improvement in oral, intestinal, and general condition symptoms among patients at facility one who used EF-2001 in granule formulation The data are presented as n (%). *Intestinal symptoms were observed in 11 patients; one patient reported both constipation and diarrhea. **Non-responders: n = 1 for improvement in oral symptoms; n=2 for willingness to continue use. The percentages were calculated based on the number of respondents. EF-2001: *Enterococcus faecalis* 2001

Parameters	Results	N (cases)	Percentage
Oral symptoms	Xerostomia	4	25
	Stomatitis	6	38
	Glossodynia	3	19
	Dysgeusia	4	25
	Tongue coating	1	6
	Halitosis	1	6
	Gingival pain	1	6
	Sticky saliva	1	6
Intestinal symptoms	Constipation	9*	56
	Diarrhea	3*	19
	Normal	5	31
Improvement in oral symptoms**	Improved	2	13
	Slightly improved	8	53
	No change	5	33
	Slightly worse	0	0
	Worse	0	0
Improvement in intestinal symptoms	Improved	3	27
	Slightly improved	2	18
	No change	6	55
	Slightly worse	0	0
	Worse	0	0
Duration until improvement of oral symptoms	1–2 weeks	3	30
	2–4 weeks	4	40
	4–8 weeks	2	20
	8-12 weeks	1	10
Duration until improvement of intestinal symptoms	1–2 weeks	3	60
	2–4 weeks	1	20
	4–8 weeks	1	20
	8-12 weeks	0	0
Willingness to continue use**	Yes	5	36
	A little	5	36
	Neither	2	14
	Not much	2	14
	No	0	0

Paste type: Oral symptoms included xerostomia (n=11, 48%), stomatitis (n=5, 22%), glossodynia (n=5, 22%), tongue coating (n=4, 17%), halitosis (n=3, 13%), dysgeusia (n=2, 9%), and gingival pain (n=1, 4%), with xerostomia being the most common. Intestinal symptoms included constipation (n=19, 83%), diarrhea (n=4, 17%); one was normal (4%). The oral symptom improvement rate was 75% (six (30%) “improved,” nine (45%) “slightly improved,” five (25%) “no change,” zero (0%) “slightly worse,” and zero (0%) “worse"). Time to improvement was one to two weeks (n = 4, 27%), two to four weeks (n = 5, 33%), one to two months (n = 4, 27%), and two to four months (n = 1, 7%).

The improvement rate for intestinal symptoms was 66% (seven (33%) “improved,” seven (33%) “slightly improved,” six (29%) “no change,” one (5%) “slightly worse,” and zero (0%) “worse”). Time to improvement was one to two weeks (n = 4, 29%), two to four weeks (n = 5, 36%), four to eight weeks (n = 4, 29%), and eight to 12 weeks (n = 1, 7%). Improvement in general condition was reported in 47% of participants (five (22%) “improved,” six (26%) “slightly improved,” 12 (52%) “no change,” zero (0%) “slightly worse,” and zero (0%) “worse”). Willingness to continue was reported by 82% (seven (30%) “yes,” 12 (52%) “a little,” two (9%) “neither,” one (4%) “not much,” one (4%) “no”) (Table 13).

**Table 13:** Self-reported improvement in oral, intestinal, and general condition symptoms among patients at facility one who used EF-2001 paste formulation. The table summarizes improvement rates, time to improvement, and participant willingness to continue EF-2001, with the paste formulation showing higher preference and efficacy in this cohort. The data are presented as n (%). *Intestinal symptoms were observed in 21 patients; two patients reported both constipation and diarrhea. **Non-responders: n = 3 for improvement in oral symptoms. The percentages were calculated based on the number of respondents. EF-2001: *Enterococcus faecalis *2001

Parameters	Results	N (cases)	Percentage
Oral symptoms	Xerostomia	11	48
	Stomatitis	5	22
	Glossodynia	5	22
	Dysgeusia	2	9
	Tongue coating	4	17
	Halitosis	3	13
	Gingival pain	1	4
	Sticky saliva	0	0
Intestinal symptoms	Constipation*	19	83
	Diarrhea*	4	17
	Normal	2	9
Improvement in oral symptoms**	Improved	6	30
	Slightly improved	9	45
	No change	5	25
	Slightly worse	0	0
	Worse	0	0
Improvement in intestinal symptoms	Improved	7	33
	Slightly improved	7	33
	No change	6	29
	Slightly worse	1	5
	Worse	0	0
Duration until improvement of oral symptoms	1–2 weeks	4	29
	2–4 weeks	5	36
	4–8 weeks	4	29
	8-12 weeks	1	7
Duration until improvement of intestinal symptoms	1–2 weeks	4	29
	2–4 weeks	7	50
	4–8 weeks	3	21
	8-12 weeks	0	0
Willingness to continue use	Yes	7	30
	A little	12	52
	Neither	2	9
	Not much	1	4
	No	1	4

Facility Two

Granule type: Reported oral symptoms included glossodynia (n=11, 46%), xerostomia (n=8, 33%), tongue coating (n=8, 33%), stomatitis (n=7, 29%), halitosis (n=6, 25%), gingival pain (n=4, 17%), dysgeusia (n=3, 13%), and sticky saliva (n=3, 13%), with stomatitis being the most frequent. Intestinal symptoms included constipation (n=11, 46%), diarrhea (n=4, 17%); nine were normal (38%). A total of 63% (15/24) reported at least one intestinal symptom.

The oral symptom improvement rate was 54% (six (25%) “improved,” seven (29%) “slightly improved,” 11 (46%) “no change,” zero (0%) “slightly worse,” and zero (0%) “worse”). Time to improvement was one to two weeks (n = 2, 15%), two to four weeks (n = 6, 46%), four to eight weeks (n = 4, 31%), and eight to 12 weeks (n = 1, 8%). The improvement rate for intestinal symptoms was 60% (two (13%) “improved,” seven (47%) “slightly improved,” six (40%) “no change,” zero (0%) “slightly worse,” and zero (0%) “worse”). Time to improvement was one to two weeks (n = 1, 11%), two to four weeks (n = 4, 44%), four to eight weeks (n = 4, 44%), and eight to 12 weeks (n = 0, 0%). Willingness to continue treatment was reported by 96% (11 (46%) “yes,” 12 (50%) “a little,” zero (0%) “neither,” one (4%) “not much,” and zero (0%) “no”) (Table 14).

**Table 14:** Self-reported improvement in oral and intestinal symptoms among patients at facility two who used EF-2001 granule formulation The data are presented as n (%). EF-2001: Enterococcus faecalis 2001

Parameters	Results	N (cases)	Percentage
Oral symptoms	Xerostomia	8	33
	Stomatitis	7	29
	Glossodynia	11	46
	Dysgeusia	3	13
	Tongue coating	8	33
	Halitosis	6	25
	Gingival pain	4	17
	Sticky saliva	3	13
Intestinal symptoms	Constipation	11	46
	Diarrhea	4	17
	Normal	9	38
Improvement in oral symptoms	Improved	6	25
	Slightly improved	7	29
	No change	11	46
	Slightly worse	0	0
	Worse	0	0
Improvement in intestinal symptoms	Improved	2	13
	Slightly improved	7	47
	No change	6	40
	Slightly worse	0	0
	Worse	0	0
Duration until improvement of oral symptoms	1–2 weeks	2	15
	2–4 weeks	6	46
	4–8 weeks	4	31
	8-12 weeks	1	8
	12- weeks	0	0
Duration until improvement of intestinal symptoms	1–2 weeks	1	11
	2–4 weeks	4	44
	4–8 weeks	4	44
	8-12 weeks	0	0
	12- weeks	0	0
Willingness to continue use	Yes	11	46
	A little	12	50
	Neither	0	0
	Not much	1	4
	No	0	0

Paste type: Oral symptoms included xerostomia (n=36, 48%), stomatitis (n=27, 36%), glossodynia (n=25, 33%), dysgeusia (n=8, 11%), tongue coating (n=20, 27%), halitosis (n=13, 17%), gingival pain (n=14, 19%), and sticky saliva (n=11, 15%), with xerostomia being most common. Intestinal symptoms included constipation (n=40, 53%) and diarrhea (n=12, 16%); 26 (35%) were normal, with 69% (52/75) reporting at least one symptom. The oral symptom improvement rate was 68% (21 (28%) “improved,” 30 (40%) “slightly improved,” 16 (33%) “no change,” zero (0%) “slightly worse,” and zero (0%) “worse”). Time to improvement was one to two weeks (n = 9, 18%), two to four weeks (n = 13, 25%), four to eight weeks (n = 11, 22%), eight to 12 weeks (n = 13, 25%), and >12 weeks (n = 5. 10%). The improvement rate for intestinal symptoms was 67% (11 (22%) “improved,” 22 (45%) “slightly improved,” 16 (33%) “no change,” zero (0%) “slightly worse,” and zero (0%) “worse”). Time to improvement was one to two weeks (n = 5, 15%), two to four weeks (n = 9, 27%), four to eight weeks (n = 11, 33%), eight to 12 weeks (n = 7, 21%), and >12 weeks (n = 1, 3%). Willingness to continue was reported by 75% (28 (38%) “yes,” 28 (38%) “a little,” nine (12%) “neither,” eight (11%) “not much,” one (1%) “no”) (Table 15).

**Table 15:** Self-reported improvement in oral and intestinal symptoms among patients at facility two who used EF-2001 paste formulation Improvement trends, symptom resolution timelines, and treatment satisfaction are provided, showing similar efficacy to the granule form but a stronger preference among paste users. The data are presented as n (%). *Intestinal symptoms were observed in 52 patients; three patients reported both constipation and diarrhea.

Parameters	Results	N (cases)	Percentage
Oral symptoms	Xerostomia	36	48
	Stomatitis	27	36
	Glossodynia	25	33
	Dysgeusia	8	11
	Tongue coating	20	27
	Halitosis	13	17
	Gingival pain	14	19
	Sticky saliva	11	15
Intestinal symptoms	Constipation	40	53
	Diarrhea	12	16
	Normal	26	35
Improvement in oral symptoms	Improved	21	28
	Slightly improved	30	40
	No change	24	32
	Slightly worse	0	0
	Worse	0	0
Improvement in intestinal symptoms	Improved	11	22
	Slightly improved	22	45
	No change	16	33
	Slightly worse	0	0
	Worse	0	0
Duration until improvement of oral symptoms	1–2 weeks	9	18
	2–4 weeks	13	25
	4–8 weeks	11	22
	8-12 weeks	13	25
	12- weeks	5	10
Duration until improvement of intestinal symptoms	1–2 weeks	5	15
	2–4 weeks	9	27
	4–8 weeks	11	33
	8-12 weeks	7	21
	12- weeks	1	3
Willingness to continue use	Yes	28	38
	A little	28	38
	Neither	9	12
	Not much	8	11
	No	1	1
